# A Review on CAD/CAM Yttria-Stabilized Tetragonal Zirconia Polycrystal (Y-TZP) and Polymethyl Methacrylate (PMMA) and Their Biological Behavior

**DOI:** 10.3390/polym14050906

**Published:** 2022-02-24

**Authors:** Cristina Herráez-Galindo, María Rizo-Gorrita, Serafín Maza-Solano, María-Angeles Serrera-Figallo, Daniel Torres-Lagares

**Affiliations:** Faculty of Dentistry, University of Seville, Avicena Street, 41009 Seville, Spain; crisnach.15@gmail.com (C.H.-G.); mrizo@us.es (M.R.-G.); serafinmazasolano@gmail.com (S.M.-S.); danieltl@us.es (D.T.-L.)

**Keywords:** gingival fibroblast, computer-aided design/computer-aided manufacturing (CAD/CAM) materials, yttria-stabilized tetragonal zirconia polycrystal (Y-TZP), polymethyl methacrylate (PMMA)

## Abstract

Yttria-stabilized tetragonal zirconia polycrystal (Y-TZP) and polymethyl methacrylate (PMMA) are used very often in dentistry. Y-TZP is the most widely used zirconia dental ceramic, and PMMA has classically been used in removable prosthesis manufacturing. Both types of materials are commercialized in CAD/CAM system blocks and represent alternatives for long-lasting temporary (PMMA) or definitive (Y-TZP) implantological abutments. The aim of the present work is to reveal that human gingival fibroblasts (HGFs) have a favorable response when they are in contact with Y-TZP or PMMA as a dental implant abutment or implant-supported fixed prosthesis, and also to review their principal characteristics. We conducted an electronic search in the PubMed database. From an initial search of more than 32,000 articles, the application of filters reduced this number to 5104. After reading the abstracts and titles, we reduced the eligible articles to 23. Ultimately, we have included eight articles in this review.

## 1. Introduction

Implant placement is followed by the osseointegration progress, after which a second surgery phase occurs [1,2,3,4,5,6]. During this process, soft and bone tissues are healing around the implant and transepitelial surface, respectively. Implant abutment connects the internal and external oral environments; therefore, soft tissue creates a hermetic barrier around the abutment to prevent bacteria crossing this area and affecting clinical implant success [3,4,5,7,8,9,10]. Keratinocytes and fibroblasts are the main cells in charge of soft tissue sealing around the dental implant abutment; they prevent apical migration of the junctional epithelium and bone resorption [4,5,11] and reduce bacteria adhesion to the implant-abutment junction [12,13].

When selecting an implant material, it is important to take its biocompatibility into account as well as its cellular behavior around these surfaces [4]. A computer-aided design/computer-aided manufacturing (CAD/CAM) system has promoted the implantology evolution. This technology, introduced in 1985, helps clinicians decrease production time and adapt materials and structures [14,15,16,17,18,19,20,21]. Many new-generation dentistry materials are milled with this system, among which are Y-TZP and PMMA.

Zirconium has favorable mechanical properties as well as high biological stability and biocompatibility, and its surface has low plaque retention. The principal disadvantage of this material is its opacity (less aesthetic than other ceramic materials), which is solved when stabilized with yttrium. Y-TZP represents a more aesthetic option with more translucence. This material is the most common type of zirconia used in dentistry today [5,22,23,24,25,26,27,28].

This material exhibits favorable mechanical properties, largely due to the particle size in the structure (0.2–0.5 µm), which helps maintain the stable tetragonal phase. It presents a high flexural strength (900–1200 MPa), fracture resistance (7–10 MPa m1/2), and elasticity modulus (210 GPa). It is increasingly considered the alternative to titanium for aesthetic dental implant abutments in final restorations [29].

PMMA is a synthetic polymer that provides strength, color stability, and ease of repair, which are some of the essential qualities required for provisional material [4]. Some manufacturers have converted this classical removable prosthesis material into a long-term crown or abutment temporary material milled with a CAD/CAM system. This option reduces some of the principal PMMA disadvantages, as it releases monomer into the medium during polymerization because CAD/CAM allows for a controlled polymerization under optimum pressure and temperature. Similarly to reticular infiltrate, polymethyl methacrylate CAD/CAM blocks have various specifications [16,19,29,30,31,32,33,34,35,36,37].

This method also minimizes clinical chairside time and enables better marginal fit and strength. Its favorable mechanical properties, i.e., high elastic modulus (2800 MPa) and flexural resistance (>80 MPa), makes PMMA one of the most used temporary materials [30,33].

**Figure 1:**
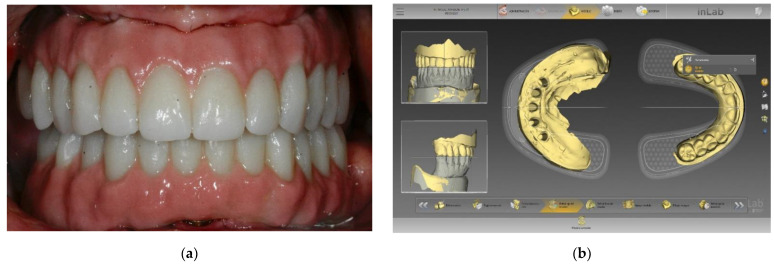
Clinical example of Y-TZP (Corcon^®^ htML Dentsply Sirona, York, PA, USA) used in an implant for definitive rehabilitation: (**a**) Frontal clinical view of the definitive prosthesis placed over five implants, (**b**) View of the design software used in this clinical case (inLab CAD Dentsply Sirona, York, PA, USA).

**Figure 2:**
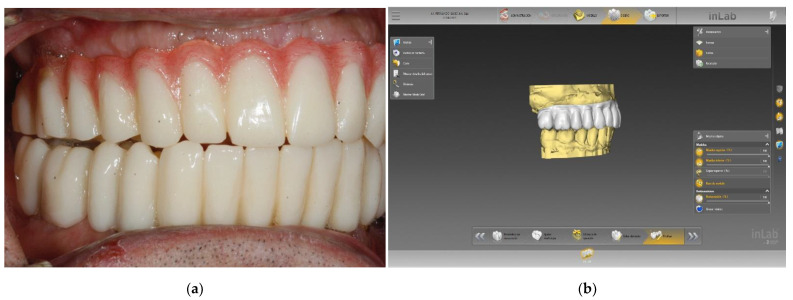
Clinical example of PMMA (Telio CAD Ivoclar-Vivadent, Schaan, Liechtenstein) used in an implant for temporary rehabilitation: (**a**) Lateral clinical view of the definitive prosthesis placed over five implants, (**b**) View of the design software used in this clinical case (inLab CAD Dentsply Sirona, York, PA, USA).

Y-TZP and PMMA are widely used in daily clinical practice, with appropriate results in most patients. Figure 1 and Figure 2 show some examples. Even though both materials have been widely studied in the last few years (especially PMMA, which has been used in dentistry for a long time), only a few authors have pointed out their CAD/CAM manufacturing and application as a crown or trasepitelial abutment and their peripheral cell contact. Furthermore, the aim of the present review is to analyze human gingival fibroblasts’ response to contact with both these materials.

## 2. Materials and Methods

We conducted the present literature review according to Preferred Reporting Items for Systematic Reviews and Meta-Analyses (PRISMA) and following the PICO format (P: population; I: intervention; C: comparison; O: outcome) in January 2022 (Table 1).

We conducted an additional electronic search in the PubMed and Scopus databases to identify both materials’ behavior (Y-TZP and PMMA) in the oral environment and in contact with peri-implant soft tissue. We used the following search strategy:

- (“yttria-stabilized tetragonal zirconia polycrystal” OR “ytzp” OR “ytrium tetragonal zirconia polycrystal” OR “pmma” OR “polymethyl methacrylate”) AND (“dental implant” OR “dentistry”)
- (“yttria-stabilized tetragonal zirconia polycrystal” OR “ytzp” OR “ytrium tetragonal zirconia polycrystal” OR “pmma” OR “polymethyl methacrylate”) AND “dentistry and restoration”
- (“yttria-stabilized tetragonal zirconia polycrystal” OR “ytzp” OR “ytrium tetragonal zirconia polycrystal” OR “pmma” OR “polymethyl methacrylate”) AND (“dental implant” OR “dental prosthesis” OR “cad cam”)

We limited the search to English and Spanish publications, reviews, systematic reviews, meta-analyses, clinical trials, clinical studies, and comparative studies. We also filtered articles obtained for those that had been published in the last 5 years (from January 2017 to January 2022).

Once we filtered the publications, we applied the inclusion (Table 2) and exclusion criteria (Table 3).

**Figure 3:**
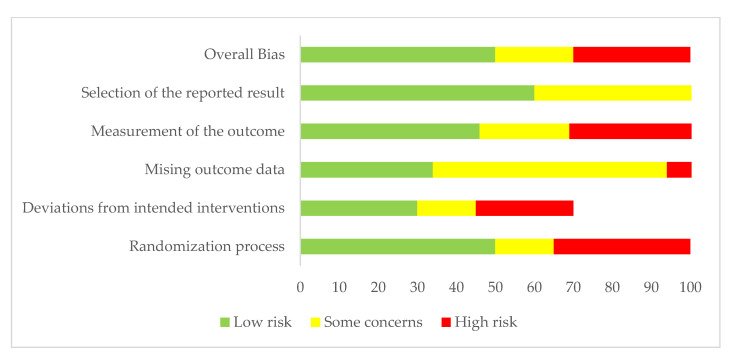
Risk of bias graph, review of authors’ judgements about each risk of bias item presented as a percentage.

Two experts conducted the paper selection, both of whom declared they did not have conflicts in this selection. Figure 3, represented as a “traffic lane” chart, presents the risk of bias for each article selected.

## 3. Results

The electronic search using PICO format in the PubMed/Medline database yielded a total of 26,057 articles. We identified 6003 more articles in the conventional electronic search on the same platform. After applying our filter (type of study and <5 years since literature search), we obtained 4461 articles in the first search and 643 in the second one.

Reviewers proceeded to screen all the studies using title and abstract; they then excluded duplicates and unavailable articles. We full-text analyzed 23 publications of interest, resulting in a total of 8 articles included in this paper.

Table 4 summarizes all selected studies.

## 4. Discussion

Gingiva is the epithelium in charge of creating a barrier (biological seal) between the abutment and the connective tissue. This barrier should adhere to the implant abutment surface, which has the function of creating stability between soft and hard tissues (protecting implant—abutment connection and peri-implant bone) and protecting against noxious bacteria; it also has an acceptable aesthetic quality. The protective barrier requires a nontoxic material that favors the attachment and growth of the surrounding tissues [29]. Y-TZP and PMMA have been widely studied, and the findings of different authors coincide in their biocompatibility and appropriate fibroblast response.

We analyzed yttria-stabilized tetragonal zirconia polycrystal and polymethyl methacrylate from a clinical perspective in two of the selected articles on which we based this research [38,39,40,41,42]. Bagegni A et al. presented a complete meta-analysis, examining various implant-fixed restorations (interim or definitive) and their effect on implant survival. On one hand, the authors concluded that metal-ceramic FCDs are more effective in implant survival than other materials [38] because they did not differentiate between groups of zirconium-derived materials. Moreover, the authors assumed that all material restoration should be fixed to a metal structure. On the other hand, they deduced that the survival of FCDs seems not to be affected by the choice of restorative material [38].

Díez-Quijano et al. compared PMMA to POM (polyoxymethylene) as a provisional implant-prosthetic material in a randomized clinical trial. Experts evaluated some clinical parameters (surface color, anatomic shape, marginal integrity, and screw-related complications) during the follow-up periods (1 week, 3 months, and 6 months). Better results were obtained in PMMA cases [42].

Classically, PMMA was manufactured as a direct or indirect polymerizable material. However, today, the CAD/CAM system has helped improve this material. PMMA CAD/CAM milling is especially useful in cases of implant-supported prosthesis and implant abutment. A number of authors agree about one of the most important advantages of this system: reduction of polymerization shrinkage and elimination of residual monomer released [4,39,40].

In some studies, researchers compared CAD/CAM PMMA specimens with various materials. All of them obtained excellent results in relation to the material cytotoxicity. They also discovered that HGFs have appropriate COL-1 production and surface attachment [29,40].

Other PMMA-surface parameters (such as roughness) were analyzed, producing similar results to those of gold standard materials [4,39,40]. These outcomes support the use of polymethyl methacrylate as an excellent interim restoration and implant-abutment material.

Y-TZP has been compared to other classical materials (such as lithium disilicate and titanium) as a dental-implant abutment. Its noteworthy physical and biological properties make this material an effective alternative to classical materials. Some of its properties are high biological stability, translucence (it does not need to be covered with feldespathic ceramics), an elastic modulus of 210 MPa, and excellent mechanical strength (800–1200 MPa) [5,29]. We carried out MTT assays with Y-TZP discs, obtaining appropriate results in in vitro studies with human gingival fibroblasts [29,43].

This material’s high flexibility and other physical alterations in the face of aging procedures have also been tested. This affirmation proves this material’s high resistance in an oral environment [41].

Researchers have proven that fibroblasts grown on Y-TZP shows great extension of the actin filaments and an elongated shape, which occurs because fibroblasts that grow on smoother surfaces (such as Y-TZP) are forced to stabilize themselves, developing a strong network of actin fibers and appearing more elongated and spread out. HGFs present a strong cytoskeleton when they grow on heterogeneous topography, such as rough surfaces [5,8,11].

Few papers have been published that analyze human gingival fibroblasts’ response to contact with Y-TZP or PMMA. Future long-term clinical or in vitro studies should be proposed to complement clinical and biological information collected in the present review.

## 5. Conclusions

Both materials have been demonstrated to be effective options for use as temporary or definitive abutment/prosthesis material. Many studies have shown that they promote a favorable fibroblast response, which translates into an appropriate soft-tissue seal, low bacteria adhesion, and long duration of the material in the oral cavity.

## Figures and Tables

**Table 1:** Overview of the search strategy following the PICO format.

PICO Question		What Are the Differences between Fibroblast Behavior on YTZP and PMMA?
Search strategy	P (Problem, population)	edentulous OR crown OR edentulism OR fixed dental prosthesis OR implant-supported prosthesis OR implant-supported denture OR dental prosthesis, implant-supported OR dental abutment
	I (Intervention)	yttria-stabilized tetragonal zirconia polycrystal OR ytzp OR y-tzp OR ytzps OR y-tzps AND cad cam
	C (Comparison)	polymethyl methacrylate OR pmma AND cad cam
	O (Outcome)	fibroblasts OR gingival fibroblast OR gingiva

**Table 2:** Search strategy and results of identification, screening for eligibility, and inclusion of publications considered for review.

Identification	Records identified through electronic database search according to PICO format (PubMed) *n* = 26,057 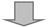	Records identified through electronic database search according to keyword combination strategy (PubMed and Scopus) *n* = 6003 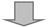	
Screening	*n* = 4461 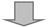	*n* = 643 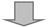	Filter application
	*n* = 17	*n* = 27	Studies screened (title and abstract)
Eligibility	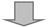 Full-text articles assessed for eligibility *n* = 23		
Included	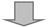 Total Studies included for quantitative synthesis *n* = 8		

**Table 3:** Inclusion and exclusion criteria list.

Inclusion Criteria	Exclusion Criteria
Publications in English or Spanish	Studies on animals
CAD/CAM Y-TZP or PMMA	Patients rehabilitated with removal prosthesis
Fixed implant prosthesis	Teeth restorations
Implant abutment	The material’s aesthetic characteristics
The material’s physical and biological characteristics	Implant material

**Table 4:** Overview of included studies.

Author, Publication Year	Study Type	Material	Implants/Discs (Total No.)	Aim	Main Conclusion
Bagegni A et al. 2019 [38]	Systematic review	Metal-ceramic	8938	Assess the influence of various restorative materials on implant survival supporting FCDs **.	Implant-supported FCD material selection seems not to affect prosthetic survival rates.
	Meta-analysis	Alloy Titanium Ceramic veneer Metal framework *			
Pituru SM et al. 2020 [39]	Review	PMMA	NR	Synthetize main PMMA characteristics as interim implant-prosthetic restoration material.	PMMA is an interim prosthetic material with predictable prosthetic results.
Shim JS et al. 2019 [40]	In vitro study	Poly(ethyl methacrylate) PMMA	210	Evaluate HGFs’ response to various interim prosthetic materials fabricated using three methods (direct, indirect, CAD/CAM).	PMMA manufactured by CAD/CAM system offers lower cytotoxicity to HGF and better cell attachment.
Herráez-Galindo C et al. 2019 [4]	In vitro study	PMMA Lithium disilicate	NR	Compare material surface and HGF behavior.	The two materials exhibited similar cellular reactions.
Guilardi LF et al. 2017 [41]	In vitro study	Y-TZP	30	Characterize and compare the effect of various aging regimens on surface characteristics, structural stability, and mechanical performance.	None of the aging regimens impaired Y-TZP’s mechanical behavior.
Díez-Quijano C et al. 2020 [42]	Randomized clinical trial	POM PMMA	49	Evaluate clinical performance of both CAD/CAM materials in implant-supported interim restorations.	PMMA performed better than POM.
Rizo-Gorrita M et al. 2019 [29]	In vitro study	PMMA LS_2_ Y-TZP ZLS	160	Evaluate cytotoxic effect and COL-1 secretion of HGFs for materials studied.	Ceramic materials showed better cell responses than polymer materials.
Pandoleon P et al. 2019 [43]	In vitro study	Y-TZP LS_2_ Ti	315	Investigate biological effect of Y-TZP abutment compared to LS_2_ and Ti and HGFs’ viability and attachment properties.	Comparable biological results in Y-TZP and conventional abutment materials.

* Implant-supported FCDs, ** FCDs (fixed completed dentures).

## Data Availability

The data presented in this study are available on request from the corresponding author.
